# Untargeted metabolomics reveals immune-metabolic signatures in established cases of rheumatoid arthritis

**DOI:** 10.3389/fmolb.2026.1755542

**Published:** 2026-02-16

**Authors:** Afshan Masood, Reem Almalki, Abeer Malkawi, Maha Al Mogren, Amal Jaafar, Hicham Benabdelkamel, Assim A. Alfadda, Amina Fallata, Abdurhman S. Alarfaj, Mohamed Siaj, Anas M. Abdel Rahman

**Affiliations:** 1 Department of Chemistry and Biochemistry, Université du Québec à Montréal (UQAM), Montreal, QC, Canada; 2 Proteomics Resource Unit, Obesity Research Center, College of Medicine, King Saud University, Riyadh, Saudi Arabia; 3 Metabolomics Section, Precision Medicine Laboratory Department, Genome Medicine Center of Excellence, King Faisal Specialist Hospital and Research Centre (KFSHRC), Riyadh, Saudi Arabia; 4 Department of Medicine, College of Medicine, King Saud University, Riyadh, Saudi Arabia; 5 Strategic Center for Diabetes Research, College of Medicine, King Saud University, Riyadh, Saudi Arabia; 6 Rheumatology Unit, Department of Medicine, College of Medicine, King Saud University, Riyadh, Saudi Arabia; 7 Department of Biochemistry and Molecular Medicine, College of Medicine, Alfaisal University, Riyadh, Saudi Arabia

**Keywords:** glycerophospholipids, immune–metabolic dysregulation, metabolomics, N2-acetyl N6-methyllysine, nucleic acids, plasma biomarkers, rheumatoid arthritis

## Abstract

**Introduction:**

Rheumatoid arthritis (RA) is a complex progressive autoimmune disorder wherein chronic inflammation is tightly coupled to metabolic reprogramming. The known diagnostic markers are not sensitive and specific enough to reflect disease activity. Finding a metabolomics-based biomarker specific for established cases of RA is important. This study aimed to investigate the metabolomic profiles of patients with established RA compared to those of controls.

**Methods:**

An untargeted high resolution mass spectrometry (MS)-based metabolomics approach with bioinformatics analysis was used to analyze 122 plasma samples, patients (n = 60), and controls (n = 62).

**Results:**

A total of 300 significantly dysregulated metabolites (unpaired t-test with FDR q value < 0.05, FC cut off 1.5) were identified between RA and controls, where 147 were upregulated and 153 downregulated. From among these, 182 metabolites were identified and annotated and after excluding the exogenous metabolites 60 endogenous metabolites were successfully identified. Results from the OPLSDA model showed a clear separation between patients with RA and controls (Q2 = 0.736, R2 = 0.988), indicating significant metabolic differences between the groups. The plasma metabolomics profile revealed statistically significant changes in metabolites belonging to different classes including those involved in lipid (including Succinyladenosine, CDP- DG (PGE_2_/i-19: 0), PGP (i-24:0/PGD2), Octadecenoylcarnitine), amino acid (including L-Isoleucine, Cysteinyl-Serine), and nucleotide (Inosine, N6-Methyladenosine), metabolisms in RA patients, consistent with immune–metabolic dysregulation. Bioinformatics and network pathway analysis using IPA showed interconnectedness between the metabolites centered around IL-6, IL-2, IL-1, MAPK, and kininogen. The pathways most affected between RA and controls included humoral immune response, inflammatory response, hematological system development, and function. The identified metabolites influenced eicosanoid/kinin signaling, nucleic acid–mediated innate immune activation, mitochondrial dysfunction, and altered glycosylation. Subgroup analysis based on stratification using erythrocyte sedimentation rate (ESR) above 35 mm/h identified 67 metabolites that differentiated patients with high versus low ESR. Among these, three metabolites, namely, cysteinyl-serine (upregulated), tyrosyl-arginine and N2-acetyl N6-methyllysine (downregulated) overlapped with the metabolites identified in the comparison between RA and controls, suggesting links between specific metabolic changes and systemic inflammation.

**Conclusion:**

Our findings support the potential of plasma metabolomics for phenotyping and highlight potential candidate biomarkers for disease prognosis and monitoring in RA.

## 1 Introduction

Rheumatoid arthritis (RA) is a complex systemic autoimmune disorder that extends beyond being a joint disease. RA is characterized by joint inflammation with pain and swelling, which can lead to irreversible cartilage and bone damage. The etiology is still unknown, but environmental factors, a genetic predisposition, and autoimmunity are involved with the disease’s susceptibility and severity (Scott et al., 2010). The pathophysiology of RA results from the activation of self-reactive T and B cells, which leads to synovitis, cellular infiltration, and a disorganized process of bone destruction and remodeling. The joint space is lined by a synovial membrane that suffers a tumor-like enlargement, called pannus, with local destructive effects (Choy, 2012). While joint inflammation (articular manifestations) is a hallmark of RA, it is also considered as a systemic disease with extrarticular manifestations affecting various organs. This leads to an increased risk in development of a range of complications and comorbidities such as osteoporosis, cardiovascular disease, diabetes, infections, and certain types of cancer (van Onna and Boonen, 2016). In clinical practice, it is often noted that patients with long standing RA on treatment present with minimal or no symptoms and are clinically in remission, generally feel unwell and experience residual pains, the underlying factor for which are still unclear (Motyl et al., 2024).

Although established serological markers of diagnosis, such as rheumatoid factor and anti-cyclic citrullinated peptide antibodies, markers of inflammation such as ESR and CRP, are commonly used in clinical practice for early diagnosis, disease stratification, but long-term monitoring remains challenging. These limitations are evident in patients who achieve clinical remission based on joint scores but continue to show elevated markers of inflammation, indicating ongoing subclinical inflammation and increasing the risk of relapse or joint damage (Morales-Ivorra et al., 2022).

Omics studies, including metabolomics, have emerged as a powerful tool that helps in unravelling the biochemical basis of different diseases, including RA. Unlike genomics and proteomics, which capture partially dynamic layers of disease biology, metabolomic profiling reflects the real-time biochemical consequences of cellular activity, immune activation, and therapeutic intervention (Qiu et al., 2023). In our previous study we identified significant changes in proteins between RA and control groups (Masood et al., 2025). At the molecular level, metabolomics gives dynamic insights into inflammatory, immunologic, and energy-related perturbations. It also provides information that can be predictive of distinctive phenotypes within the disease. In systemic diseases such as RA, alterations in the circulating metabolomic profile may arise from an interplay of genetic susceptibility, localized inflammatory activity, comorbid conditions, and environmental influences (Coras et al., 2020). Recent metabolomics studies have identified altered pathways in RA, including elevated levels of amino acids like glutamine, tryptophan, and arginine; perturbed tricarboxylic acid cycle intermediates; dysregulated lipid mediators such as prostaglandins, lysophospholipids, and nucleotides (Xu et al., 2022; Kishikawa et al., 2021). Over the last decade, untargeted and targeted metabolomics have consistently shown broad biochemical dysregulation in RA and links to clinical activity and outcomes. In plasma, Hur et al. (2021) identified metabolite signatures that correlated strongly with DAS28-CRP and improved quantitative disease-activity prediction using multivariable models, establishing metabolomics as a viable tool for precision phenotyping (Hur et al., 2021). Koh et al. (2022) demonstrated that synovial fluid lipidomes are profoundly perturbed and that serum lipid profiles distinguish active RA from remission and even preclinical RA, tying lipid remodeling to disease evolution (Koh et al., 2022). Integrative studies have combined plasma metabolomics with the gut microbiome, revealing alterations in glycerophospholipid and amino-acid pathways that discriminate RA from controls and highlight gut–joint metabolic crosstalk (Zhu et al., 2022). In early RA, large-cohort serum profiling has linked baseline metabolites (organic acids and nucleosides) to subsequent disease activity and treatment response, enabling predictive modeling, albeit with moderate performance (Fatima et al., 2025). Metabolomics was also used to reveal the changes within the synovial fluid that are uniquely present in the joint environment and not observed in circulation (serum or plasma) (Carlson et al., 2019). However, most of these studies focus on early or active RA stages, with limited emphasis on patients with minimal or no symptoms who may still harbor low-grade inflammation.

Recent advances in metabolomics have shifted the paradigm of RA research, enabling the identification of disease-specific metabolomic patterns and signatures across different phenotypes. Therefore, we conducted an untargeted, high-resolution plasma metabolomics study with bioinformatics and network pathway analysis comparing established RA patients to controls to define the systemic metabolic signature of the disease. As a secondary analysis, we stratified patients by ESR to investigate whether specific metabolite patterns are related with residual inflammatory burden.

## 2 Materials and methods

### 2.1 Ethical approval, patient recruitment, and consent to participate

The study procedures and protocols were reviewed and approved by the institutional review board of the College of Medicine, King Saud University. Written informed consent was obtained from all the participants (IRB number: E-21-6341). The primary treating physician determined the diagnosis for RA based on the American College of Rheumatology and the European League Against Rheumatism (ACR/EULAR) 2010 criteria. The patients in the present study were recruited from those attending the rheumatology outpatient clinics at King Khalid University Hospital, College of Medicine, King Saud University, in the age group of 36–65 years. A total of 60 patients with known RA for a mean duration of 20 years mostly women, were recruited. The patients at the time of the clinical visit did not have any symptoms of early morning stiffness, tender or swollen joints and were considered to be in clinical remission with a (Disease Activity Score-28 (DAS28) <2.6). Control plasma from age-matched volunteers who were otherwise healthy was collected after informed consent was obtained. The baseline demographic information and clinical laboratory parameters, including complete blood count with an ESR, lipid markers, liver function tests, fasting glucose, glycated hemoglobin, and C-reactive protein (CRP), were collected from the patient’s medical records (Table 1). Patients with RA were next stratified according to their ESR, levels >35 mm/h were considered as high and below that considered as low. Additional stratification was carried out using CRP, levels > 10 mg/dL being considered as high and below that considered as low. The study procedures were conducted according to the ethical standards of the Declaration of Helsinki and the International Conference on Harmonization Good Clinical Practice guidelines.

**TABLE 1:** Baseline demographic and clinical characteristics of patients with rheumatoid arthritis and the control groups.

Characteristics	Patients (mean ± std) (n = 60)	Controls (mean ± std) (n = 62)	P-value
Duration (yrs)	15 ± 7	na	na
AGE	49.88 ± 12.05	47.52 ± 13.86	0.31
Gender (F/M)	55/5	57/6	0.38
BMI (Kg/m^2^)	28.76 ± 8.09	29.89 ± 6.21	0.38
ALT (IU/L)	18.99 ± 7.06	18.04 ± 9.01	0.51
AST (IU/L)	17.07 ± 5.03	20.14 ± 10.12	0.06
Albumin (gm/L)	42.99 ± 4.14	44.03 ± 2.70	0.10
Alk Phs (IU/L)	76.09 ± 20.37	77.57 ± 30.55	0.90
Glucose (mmol/L)	5.56 ± 2.50	5.51 ± 1.28	0.90
Chol (mmol/L)	4.63 ± 0.78	4.77 ± 0.94	0.36
HDL (mmol/L)	1.47 ± 0.41	1.48 ± 0.37	0.89
LDL (mmol/L)	2.70 ± 0.80	2.99 ± 0.81	0.06
Triglycerides (mmol/L)	1.19 ± 0.74	1.27 ± 0.54	0.50
HbA1c	5.74 ± 0.54	5.71 ± 0.84	0.87
ESR	43.04 ± 20.6	5.64 ± 2.80	0.00
CRP (mg/L)	9.70 ± 7.40	1.9 ± 3.4	0.00
RF	277.70 ± 75.46		

Values are expressed as mean ± standard deviation (SD) in parentheses. A significant difference between RA, and control (independent t-test, p-value <0.05 for parametric tests). BMI: body mass index, HB: hemoglobin, WBC: white blood cells, RBC: red blood cells, PLT: platelets, ESR: erythrocyte sedimentation rate, ALT: alanine aminotransferase, AST: aspartate transaminase, Chol: Cholesterol, HDL: high-density lipoprotein, LDL: low-density lipoprotein, HbA1c: glycated hemoglobin, ESR: erythrocyte sedimentation rate, CRP: C-reactive protein. * p-Value <0.05 is considered statistically significant.

### 2.2 Sample collection, storage, and metabolite extraction

The blood samples were collected by venipuncture into EDTA-containing tubes from all participants and processed (centrifuged at 1,500 × *g* for 10 min at 4 °C) to obtain clear plasma. The plasma was aliquoted and stored at −80 °C until the day of analysis. Metabolites were extracted from plasma using our previously established protocol (Alfadda et al., 2024). Briefly, cold methanol and chloroform were added to 50 μL of plasma, followed by water and shaking. Equal volumes of chloroform and water were added before centrifugation at 10,000 × *g* for 5 min. All samples were dried using a vacuum centrifugal evaporator and stored at − 80 °C until further analysis.

### 2.3 Metabolomics mass spectrometric analysis

Metabolomics profiling was carried out utilizing untargeted metabolomics analysis via LC-HRMS, as previously reported (Sebaa et al., 2023). Metabolites were analyzed using a Waters ACQUITY UPLC system and an Xevo G2-S QTOF mass spectrometer with an electrospray ionization source (ESI) in positive and negative modes (ESI+, ESI−). The metabolites were chromatographed using an ACQUITY UPLC XSelect column (100 mm × 2.1 mm, 2.5 μm) from Waters Ltd., Elstree, United Kingdom. The mobile phases A and B (A: 0.1% formic acid in dH2O—B: 0.1% formic acid in 1:1 v/v MeOH and ACN) were pumped to the column in a gradient mode (0–16 min 95%–5% A, 16–19 min 5% A, 19–20 min 5%–95% A, 20–22 min 5%–95% A) at a flow rate of 300 μL per minute. The MS settings were as follows: the source temperature was 150 °C, the desolvation temperature was 500 °C for both modes, the capillary voltages were 3.20 kV (ESI+) or 3 kV (ESI−), the cone voltage was 40 V, the desolvation gas flow was 800.0 L/h, and the cone gas flow was 50 L/h. In MS^E^ mode, the collision energy of low and high functions was set to 10–50 V, respectively. As indicated by the seller, the mass spectrometer was calibrated with sodium formate in the 100–1,200 Da range in both ionization modes. Data-independent acquisition (DIA) was performed in continuum mode using a Masslynx™ V4.1 workstation (Waters Inc., Milford, MA, United States).

### 2.4 Data handling and processing

The raw mass spectrometry data underwent processing through a standard pipeline involving m/z and retention time alignment, peak selection, and quality-based signal filtering, using Progenesis QI v.3.0 (Waters). Multivariate analysis was performed with MetaboAnalyst v.5.0 https://www.metaboanalyst.ca/ accessed on 10 April 2025, applying median, Pareto scaling, and log transformation to normalize the data. Models such as PLS-DA and OPLS-DA were constructed, with model fitness evaluated by R2Y and Q2 metrics. Univariate differences were assessed via (unpaired t-test, FDR, q value <0.05, Fold change (FC) 1.5) using Mass Profiler Professional (MPP) v.15.0. Venn diagrams were generated with MPP, and key features were annotated against the Human Metabolome Database with emphasis on precursor mass and fragmentation; the tolerance was 5 ppm. The workflow used targeted-untargeted annotation to combine precise mass measurements (≤5 ppm) with complete isotopic pattern analysis and diagnostic MS/MS fragmentation data. The internal RT library provided additional retention-time constraints for more than 200 recurring metabolites. The system used multiple evaluation criteria to generate a single identification score which selected the simplest molecular structure without making excessive assumptions. The identification confidence followed Metabolomics Standards Initiative and Schymanski frameworks at Level 2 for high-quality spectral matches and Level 3 for tentative candidate/class identification. The LIPID MAPS shorthand system was used to identify lipids while maintaining reporting at the lowest level possible based on head-group and neutral-loss evidence (class/species without acyl positional or double-bond localization claims). Exogenous substances, including drugs and food additives, were manually excluded from the final feature list.

### 2.5 Bioinformatic and network pathway analysis

Two independent and complementary bioinformatics approaches, MetaboAnalyst 5.0 https://www.metaboanalyst.ca/ accessed on 10 April 2025, and Ingenuity Pathway Analysis (IPA, QIAGEN), were used to analyze the metabolomic data to ensure robust interpretation. MetaboAnalyst v5 was employed for statistical analysis, visualization, and pathway-based interpretation of the metabolite data using the Metabolomics Pathway Analysis (MetPA) module based on the KEGG database. Meanwhile, IPA network pathway analysis was applied to identify connections between different metabolites and provide a biological and mechanistic perspective by integrating the metabolomic data with curated biological knowledge bases.

## 3 Results

### 3.1 Clinical and biochemical data for the study participants

The demographic and biochemical characteristics of the study participants, in the discovery and validation phases, are presented in Table 1. The mean ages of the study participants in the RA and control groups were 49.88 ± 12.05 and 47.52 ± 13.86 years, respectively, and were matched in body weight, BMI, and other laboratory markers. Majority of the patients were taking methotrexate followed by sulfasalazine and only five patients were taking prednisolone as adjunct therapy. Significant differences were noted in the ESR and CRP levels, which were higher in the RA group than the controls.

### 3.2 Detection of mass ion features and metabolomics profiling between RA and control

**FIGURE 1:**
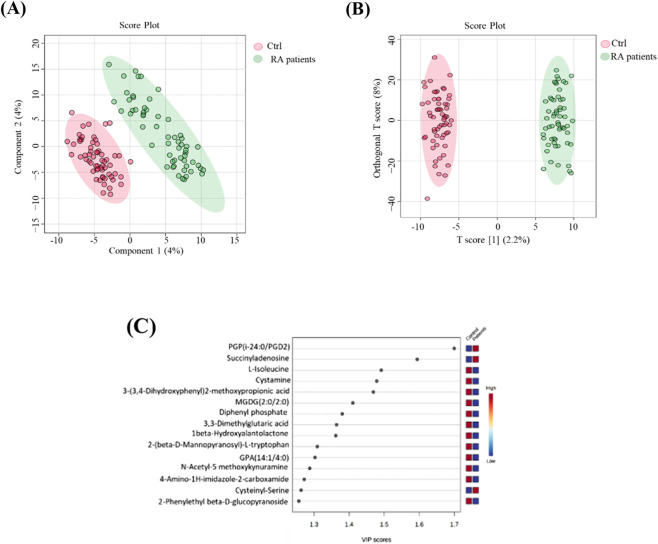
Comparison of metabolite features between the RA and control groups. **(A)** Partial least squares discriminant analysis (PLS-DA) shows separation between groups. n represents the number of metabolite features identified with a significant q-value (q < 0.05). **(B)** Orthogonal partial least squares discriminant analysis (OPLS-DA) score plot showing evident separation between RA and control groups. The robustness of the created models was evaluated by the model’s fitness (R2Y = 0.988) and predictive ability (Q2 = 0.736) values. **(C)** A variable importance in projection (VIP) score plot of discriminative metabolites in rheumatoid arthritis. Metabolites with the highest VIP scores are shown, indicating their contribution to group separation.

An initial untargeted metabolomics analysis detected a total of 18,065 mass ion features, including 11,000 in positive ion mode and 7,065 in negative ion mode. Following data preprocessing steps—such as alignment, peak picking, and removal of missing values—a filtering criterion requiring a cut-off percentage of 50% of all samples in at least one condition was applied, reducing the dataset to 12,212 features. To ensure data normality and minimize technical variation, the dataset was median-centered, log-transformed, normalized, and processed using Pareto scaling. The metabolites that distinguished between RA and control are displayed in Figure 1. The OPLS-DA, a supervised multivariate approach between the two groups, RA patients and controls, is shown in Figure 1A. The distinct separation of the RA group from the control suggests that plasma metabolites may be useful for identifying RA. The PLSDA plot also shows separation between study groups, as shown in Figure 1B.

**FIGURE 2:**
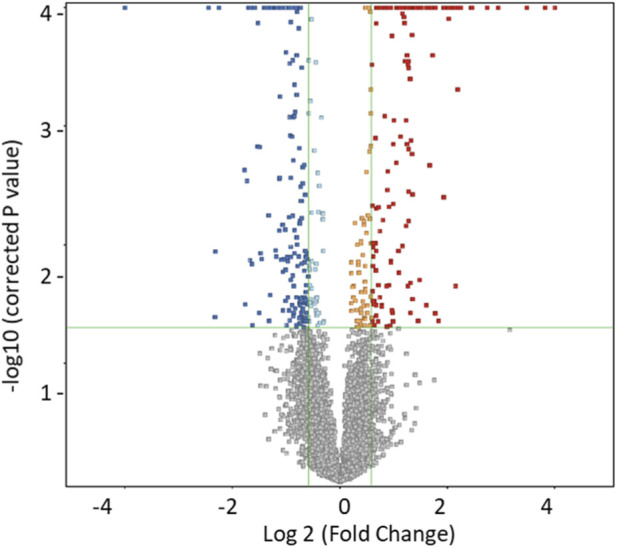
Metabolomics profiling between the RA and control groups. The volcano plot shows a significant change in the levels of several metabolites, of which red represents upregulated and blue represents downregulated tissue metabolites in RA and control groups (unpaired t-test, FDR q value <0.05, fold change cutoff 1.5).

**FIGURE 3:**
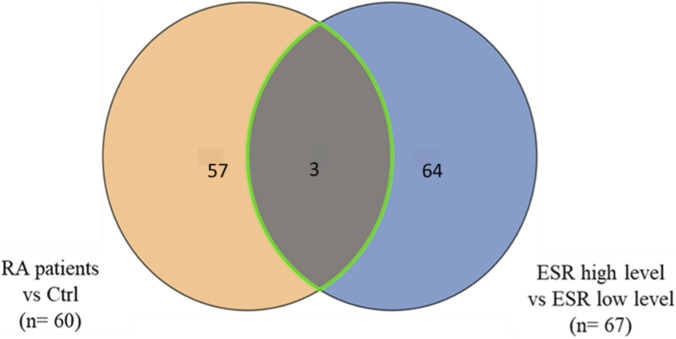
Venn diagram showing differentially expressed metabolites between study groups. Comparison of RA patients vs. healthy controls (n = 60) identified 58 unique metabolites, while comparison of RA patients with high ESR vs. low ESR levels (n = 67) revealed 65 unique metabolites. Three metabolites were found to be common to both comparisons, suggesting potential overlap between disease-associated and inflammation-associated metabolic signatures.

The statistical evaluation between two groups (RA and controls) was performed on 12,212 features using a volcano plot (unpaired T-test, FDR p < 0.05, FC 1.5), which revealed 300 significantly dysregulated metabolites between the study groups, with 147 and 153 metabolites up- and downregulated, respectively (Figure 2). Among these, these 182 were successfully annotated using Human Metabolome Database (HMDB), Metlin MS/MS, LipidBlast, lipidMap, and KEGG. After excluding the exogenous molecules (i.e., drugs, drug metabolites, environmental exposures, etc.), 60 endogenous metabolites were identified between the RA and control groups (Supplementary Table S1). The identified endogenous metabolites in the data set were cross-checked independently with our in-house library, and inosine, cytidine, l-isoleucine, mandelic acid, L-Acetylcarnitine, and 4-Hydroxyphenylpyruvic acid were confirmed. The binary subgroup analysis based on the stratification within the RA group between the high ESR compared to low ESR groups, revealed significant dysregulation in 509 metabolites with 199 and 310 metabolites being up- and downregulated, respectively. 67 endogenous metabolites were identified between patients when stratified with high and low ESR (Supplementary Table S2; Supplementary Figure S1). Of these, three endogenous metabolites were observed to overlap with the significantly dysregulated metabolites that were identified between the RA and control groups (Figure 3). Additionally, a binary comparison based on stratification using CRP levels as high vs. low revealed 6 significantly dysregulated metabolites between the groups. No overlap between these metabolites was noted with the metabolites that were significantly dysregulated in the comparison between RA and controls (60 endogenous).

### 3.3 Evaluation of metabolite biomarkers between RA and controls and bioinformatic network pathway analysis

**FIGURE 4:**
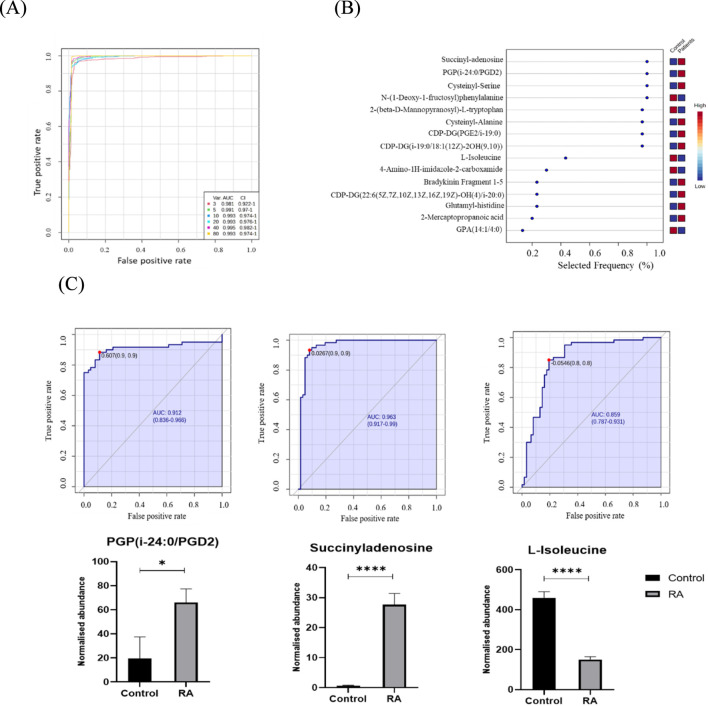
Biomarker evaluation in patients with RA and controls. **(A)** The Receiver Operating Characteristics (ROC) curve was generated by the OPLS-DA model, with Area Under the Curve (AUC) values calculated from the combination of 3, 5, 10, 20, 40, and 80 metabolites. **(B)** Frequency plot for the top 15 metabolites identified with the highest selected frequency. **(C)** Three metabolites in patients with RA had the largest AUC (FDR p < 0.05 and fold change 1.5), and their respective bar diagrams. * indicates significance <0.05 and **** indicates significance <0.0001.

To elucidate metabolic biomarkers and pathways related to RA patients, an unpaired t-test with FDR (q value < 0.1, FC cut off 1.5) was conducted and revealed 410 significantly dysregulated metabolites, where 178 and 232 metabolites were up (red) and down (blue)-regulated between the two groups. Among these, only 77 endogenous metabolites were identified. The potential biomarkers that differed between RA and controls are shown in Figure 4. OPLS-DA, a multivariate supervised method, is displayed in Figure 1A. A clear separation in the identified metabolites was observed between the groups, indicating that plasma metabolomics can be used to identify RA-related metabolites that serve as biomarkers for distinguishing RA.

A multivariate exploratory ROC analysis was conducted using OPLS-DA as a feature-ranking and classification approach based on the identified common and significantly dysregulated metabolites between the RA and control groups. The AUC of the exploratory ROC curve for the top 3 metabolites was 0.981, as shown in Figure 4A. The frequency plot shows the significantly dysregulated endogenous metabolites between the RA and control groups. OPLS-DA was used as a classification and feature ranking approach for the multivariate exploratory ROC analysis based on the common and significantly dysregulated metabolites identified between the two groups (Figure 4B). From the top 15 metabolites, two metabolites, PGP (i-24:0/PGD2) (AUC = 0.912 and 0.953), and succinyladenosine were upregulated, while expression of L-isoleucine (AUC = 0.931) was downregulated in the RA group, as shown in Figure 4C, respectively.

**FIGURE 5:**
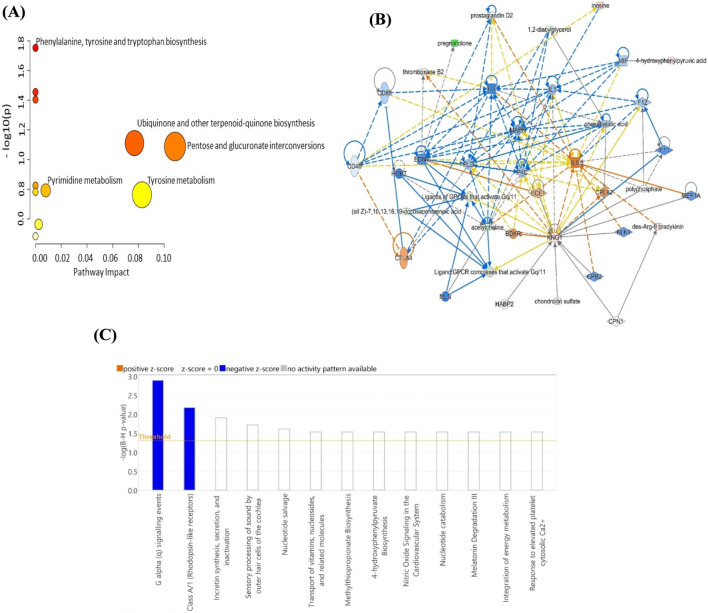
Schematic representation of the highest-scoring network pathways depicting the involvement of the differentially regulated metabolites **(A)** The impact of the metabolites on the metabolic pathways **(B)** The interactions between the metabolites with biological context with dysregulation of PKc, interleukins (1,2 and 6), kininogen as the central nodes and **(C)** The top canonical pathways ranked by the *p*-values obtained by the IPA. The interaction networks were generated through IPA (QIAGEN Inc., Hilden, Germany).

Pathway topology analysis using MetaboAnalyst revealed several significantly altered metabolic pathways in RA patients compared to controls (Figure 5A). The most enriched pathways included phenylalanine, tyrosine, and tryptophan biosynthesis, pyrimidine metabolism, and tyrosine metabolism, which are all relevant to the chronic inflammatory and immune activation state seen in RA. Additionally, the IPA generated a molecular interaction network between the metabolites and identified humoral immune response, inflammatory response, hematological system development, and function as the network pathways affected with the highest score between the RA and control groups (score of 19 and 8 focus molecules) (Figure 5B; Supplementary Table S3). The pathway suggests that RA-related metabolic changes are tightly integrated with immune and inflammatory networks, highlighting IL6, IL2, IL1, MAPK, and kininogen as central hubs, connecting metabolomics to inflammation, vascular response, and immune regulation in RA. The top canonical pathways included G alpha (q) signaling events (1.39 E^-05^, 1.8%), Class A/1 (Rhodopsin-like receptors) that were significantly enriched and predicted to be inhibited in RA patients compared to controls (z-score < 0, –log (B-H p-value) > 1.3) (Figure 5C).

## 4 Discussion

Rheumatoid arthritis (RA) is recognized not only as a joint-centered autoimmune disease but also as a systemic disorder characterized by profound metabolic reprogramming. These underlying changes in the metabolite profile can be captured by studying the plasma metabolome. Our untargeted metabolomics analysis revealed statistically significant dysregulation in levels of 77 (42 upregulated and 35 downregulated) circulating metabolites between patients with established RA and controls. The major metabolites belonged to the lipid class and included fatty acids and lipid-like molecules, nucleosides and nucleotides, amino acids, and others. These findings point to an inflammatory phenotype seen in RA and involve chronic immune activation, oxidative stress, vascular dysfunction, and tissue remodeling, all of which are central to RA pathogenesis. In this regard, elucidating the pathophysiological mechanisms and molecular signatures that drive either progression or remission in RA disease activity would be important (Aletaha and Smolen, 2018).

### 4.1 Dysregulation of plasma lipids and lipid-related metabolites with RA

The plasma lipidome in RA showed changes across multiple classes, including glycerophospholipids, ceramides, fatty acids, and acylcarnitines. Lipids are known not only as structural components but also as bioactive signaling molecules, and their systemic imbalance contributes to both local joint damage and systemic comorbidities in RA (Park et al., 2021). It is well known that inflammation underlies the pathophysiology of RA and involves changes in innate and adaptive immune systems and their cells. Multiple cell types, including T cells, B cells, dendritic cells, neutrophils, macrophages, and fibroblast-like synoviocytes, actively contribute to disease activity and progression. Previous studies indicate that lipid metabolism is significantly altered in RA by regulating the activity and function of these cells (Lei et al., 2023). In our analysis, we found notable dysregulation in lipid metabolites, with 19 metabolites decreased and 20 increased. The most substantial changes occurred in glycerophosphoglycerols (PGP), phosphatidic acid (PA), monogalactosyl diacylglycerols (MGDG), diglycerides (DG), phosphatidylethanolamine (PE), ceramides, acylcarnitines, and various fatty acids and sterol derivatives.

Phospholipids, especially glycerophospholipids, are integral to membrane architecture and function as precursors for potent lipid mediators that orchestrate immune activation, inflammation, and tissue remodeling in RA. They are also active precursors and carriers for inflammatory mediators. In our study, levels of several phosphatidylglycerol-containing prostanoid species, PGP (i-24:0/PGD_2_), and PGP(PGE_2_/i-21:0), were decreased, and the level of PGP(PGF_1_α/20:1 (11Z)) was increased. In RA, PGD2 has been shown to suppress Th1-mediated inflammation and also contributes to eosinophilic inflammation and mast cell activation. The conjugation of PGD2 to phospholipids such as PGP may enhance its bioavailability and stability in synovial fluid. Elevated levels of PGP (24:0/PGD2) in RA patients may reflect active lipid mediator signaling and remodeling of membrane phospholipids—a common feature in chronically inflamed joints (Kim et al., 2023). The dysregulation of these lipid species indicates alterations in arachidonic acid metabolism, which is known to be involved in inflammation and RA (Wang et al., 2021; Sano et al., 2020). Similarly, phosphatidic acid, PA (8:0/20:3 (8,11,14)-2OH(5,6)) was dysregulated, highlighting their role as intermediates in both glycerophospholipid biosynthesis and intracellular signaling, which regulate immune cell activation and cytokine production (Fang et al., 2001). We also observed an increase in phosphatidylethanolamine PE (PGE_2_/22:6 (4Z,7Z,10Z,13Z,16Z,19Z)), suggesting coupling between inflammatory prostaglandin synthesis and omega-3 fatty acid–derived lipid mediators, potentially reflecting a mixed pro- and resolution-phase lipid signaling environment. Collectively, these lipidomic changes support the concept that in RA, glycerophospholipid remodeling not only reflects heightened immune activation but also directly contributes to the propagation and persistence of synovial inflammation.

PAs are central intermediates in glycerophospholipid biosynthesis and serve as precursors for DG and CDP-diacylglycerols (CDP-DGs). Their elevation in plasma suggests enhanced membrane turnover and lipid signaling activity in circulating immune cells, which may contribute to the chronic inflammatory state characteristic of RA. Notably, CDP-DG species exhibited mixed expression patterns. In contrast, CDP-DG species, such as CDP-DG (22:6-OH/i-20:0) and CDP-DG (20:5-3OH/i-12:0), were significantly downregulated, suggesting impaired systemic availability or utilization of anti-inflammatory ω-3 polyunsaturated fatty acids (Duchez et al., 2019). These shifts point to a pro-inflammatory plasma lipid profile favoring eicosanoid synthesis while limiting resolution mediators. In contrast, phosphatidylinositols and platelet-activating factor (PAF) were decreased, indicative of reduced anti-inflammatory lipid signaling. These shifts collectively represent an eicosanoid-dominant, pro-inflammatory lipid remodeling in the plasma compartment of RA.

In addition to these, we identified multiple carnitine species that exhibited differential expression, with significant upregulation of octadecenoylcarnitine, and L-acetylcarnitine and a downregulation of 13-(3,4-dimethyl-5-pentylfuran-2-yl)tridecanoylcarnitine, revealing distinct perturbations in fatty acid transport and mitochondrial metabolism in RA. Alterations in carnitine metabolism in RA reflect a broader disruption in systemic energy homeostasis and immune cell metabolism. Carnitines are essential for the mitochondrial β-oxidation of long-chain fatty acids, serving as shuttles that transport acyl groups across the mitochondrial membrane. They play a central role in mitochondrial fatty acid oxidation, energy homeostasis, and immunometabolic regulation, and their dysregulation in the plasma of RA patients indicates the presence of a systemic pathophysiology (Xiang et al., 2025). The striking elevation of long-chain acylcarnitines, particularly octadecenoylcarnitine, likely reflects an imbalance between lipid influx and mitochondrial processing capacity. Long-chain acylcarnitines have been noted to increase Th17-associated cytokine production in T cells due to compromised β oxidation, which in turn promotes inflammation (Nicholas et al., 2019). Accumulation of long-chain acylcarnitines may indicate incomplete fatty acid oxidation, a metabolic phenotype associated with inflamed immune cells and mitochondrial stress. This metabolic inflexibility is consistent with reports of impaired oxidative phosphorylation and elevated glycolytic dependency in RA T cells and macrophages (Weyand et al., 2017). An increase in the levels of circulating long-chain acylcarnitines has also been found in other chronic inflammatory diseases, including chronic heart failure patients (Ahmad et al., 2016). Mitochondrial inefficiency and impaired fatty acid oxidation may contribute to systemic energy deficits. At the same time, elevations in L-acetylcarnitine, a medium-chain acylcarnitine, could signal a shift towards glycolysis and acetyl-CoA overflow (Izzo et al., 2023), consistent with the metabolic reprogramming seen in activated immune cells (Andonian et al., 2022). In animal models, L-acetyl carnitine was found to inhibit the expression of inflammatory factors and antioxidants to suppress the development of atherosclerosis (Wang et al., 2020). Our findings are in line with et al., who identified increase in seven acylcarnitine metabolites with lower disease activity (Hur et al., 2021).

The significant changes in lipid metabolites in our study underscore inflammation-driven metabolic reprogramming, oxidative stress, and immune dysregulation linked to RA. These findings demonstrate the profound reorganization of lipid metabolism in RA, aligning with previous multi-omics studies that connect lipid disturbances to immune dysregulation, synovial inflammation, and joint destruction.

### 4.2 Dysregulation of amino acids and peptides related metabolites with RA

Amino acids serve essential roles in immune cell metabolism, inflammation, and tissue remodeling. In RA patients, we observed significant perturbations in crucial amino acids, bioactive peptides, post-translationally modified residues, and oxidative derivatives, pointing toward interconnected biochemical pathways that sustain inflammation and joint damage. Changes in the levels of amino acids have been linked to abnormal immune response in RA. Previous metabolomic studies suggest a direct link between amino acid metabolism and T cell and macrophage responses by promoting and modulating inflammation, which could be involved in RA pathogenesis (Ren et al., 2017). Notably, in our study, branched-chain amino acids (BCAAs), such as L-isoleucine, were markedly decreased. BCAAs are critical energy substrates for immune cells, and their depletion suggests increased consumption by inflammatory leukocytes, especially activated macrophages and lymphocytes, which rely on these amino acids to support anabolic pathways necessary for cell proliferation and inflammatory mediator synthesis (Dimou et al., 2022). Statistically significant increases in N2-acetyl, N6-methyllysine, a double-modified product of lysine acetylation and methylation, were observed between patients with RA and controls. Lysine is an essential amino acid that has been implicated in RA pathogenesis. Post-translational modifications of lysine are abundant in histones and transcriptional regulators and are key determinants of gene expression. Elevated circulating levels likely arise from increased turnover of nuclear proteins in activated immune and synovial cells. This is in concert with the fact that RA is characterized by persistent epigenetic reprogramming, particularly histone acetylation and methylation that locks fibroblast-like synoviocytes into a pro-inflammatory, tissue-invasive phenotype (Yang et al., 2022).

We additionally identified elevated levels of 2-(β-D-mannopyranosyl)-L-tryptophan (C-mannosyl-tryptophan), a C-glycosylated post-translational modification of tryptophan. C-mannosylation in the endoplasmic reticulum is known to play critical roles in the folding, sorting, and/or secretion of substrate proteins. It is produced in part by degradation of C-mannosylated proteins through the autophagic pathway in cells and is considered a product of protein C-mannosylation turnover (Minakata et al., 2021). Previous studies have shown that it is a circulating biomarker for kidney function, peripheral artery disease, and ovarian cancer. An interesting finding is that the C-mannosyl-tryptophan modification is important for, be required for the intracellular transport of IL-21R (Siupka et al., 2015). IL-21 is a key cytokine involved in the activation and proliferation of B cells and T cells, including Th17 cells and follicular helper T cells, both of which are implicated in RA pathogenesis. IL-21 can promote inflammation by increasing the production of other pro-inflammatory cytokines and by impairing the function of regulatory T cells, and contributes to the inflammation and tissue damage characteristic of the disease (Shbeer and Ahmed, 2024). Studies have shown that IL-21 levels correlate with disease activity and radiographic damage in early RA (Dinesh and Rasool, 2018; Rasmussen et al., 2010). Levels of cystathionine sulfoxide and S-(3-oxo-3-carboxy-n-propyl) cysteine, which are both derivatives of cystathionine, an intermediate compound in the transsulfuration pathway, were decreased in patients with RA compared to controls. These are sulfur-containing amino acids formed through the oxidation of cystathionine by ROS. These compounds are intermediates in the methionine–cysteine–glutathione pathway, a central axis for redox balance (Mateen et al., 2016; Pajares et al., 2015). The decrease in their levels points to increased evidence of oxidative stress in patients with RA.

Beyond metabolic and oxidative stress pathways, our findings also implicate the neuroimmune interface in RA. We observed decreased levels of O-acetylcholine, a key neurotransmitter in the cholinergic anti-inflammatory pathway. Acetylcholine inhibits NF-κB activation and reduces pro-inflammatory cytokine release from immune cells (Tracey, 2002). Its suppression may remove an important control on systemic inflammation. On the other hand, we also observed an elevation in the levels of dynorphin B (10–13), a breakdown metabolite from the dynorphin metabolic pathway. An increase in its levels points to activation of the endogenous opioid system that may provide short-term analgesia in chronic pain syndromes and RA (Millan, 2002). Dynorphin acts on opioid receptors to produce analgesia, but it also has non-opioid effects, including activating bradykinin receptors. Bradykinin receptors (B1 and B2) are involved in inflammatory pain and hyperalgesia. Interestingly, our metabolomic analysis also identified increased levels of bradykinin and bradykinin fragments in our data set, in line with previous studies (Vanarsa et al., 2020).

Plasma levels of the dipeptides cysteinyl-serine, cysteinyl-alanine, phenylalanylvaline, and tyrosyl-arginine were increased while Arginylarginine levels were lowered in patients with RA compared to controls. RA is known to increase levels of multiple cysteine/serine proteases, which lead to heightened extracellular proteolysis, in turn generating low-molecular-weight circulating peptides. This aligns with RA peptidomics studies showing an expanded low-molecular-weight peptide/dipeptide signature in serum/plasma compared with controls. Tyrosylarginine is a dipeptide which was identified as an opiod with analgesic properties. A decrease in its levels in RA patients suggests the heightened pain response in these patients (Ueda, 2021). A decrease in arginylarginine may reflect increased consumption of L-arginine by inducible nitric-oxide synthase in activated myeloid and stromal cells, in line with previous studies that documented robust NO/iNOS signaling in RA.

### 4.3 Dysregulation of nucleotides, nucleosides, and related metabolites with RA

We found several nucleotide- and nucleoside-related metabolites altered in the plasma of patients with RA. Persistent activation of immune cells due to hypoxia and chronic inflammation in RA leads to accelerated DNA and RNA turnover in metabolizing cells, influencing circulating nucleotides in plasma (Kishikawa et al., 2021). In our study, we identified intermediates of purine/pyrimidine metabolism inosine, cytidine, and AICAR, and modified nucleosides succinyladenosine, N6-Methyladenosine, and 1-methylguanine. Levels of succinyladenosine and 1-methylguanine were elevated, while the others were down. Succinyladenosine, a modified adenosine bound to succinate, sits on the adenylosuccinate lyase arm of *de novo* purine biosynthesis and accumulates when flux through this pathway is high. Adenosine and succinate are known to act as signaling molecules and modulate immune responses, with elevated levels linked to inflammatory conditions. In the setting of chronic immune activation, a rise in circulating succinyladenosine is therefore consistent with heightened purine synthesis/turnover in proliferating immune and stromal cells (Huang et al., 2024; Magni and Ceruti, 2020). Inosine, an adenosine-pathway metabolite with anti-inflammatory actions via adenosine receptors, was also reduced; lower inosine levels fit enhanced catabolism during immune activation and the loss of an adenosinergic anti-inflammatory tone. RA and other inflammatory models document inosine’s capacity to limit cytokine output through A2A/A3 signaling (Kim and Jo, 2022).

Conversely, depletion of certain nucleosides, such as inosine and cytidine, may reflect their rapid utilization during immune-cell proliferation or altered enzymatic processing, such as increased cytidine deaminase activity. Several intermediates with immunoregulatory potential were lower. AICAR, an intermediate of *de novo* purine synthesis, was decreased, compatible with brisk flux toward IMP and a relative loss of AMPK control that otherwise restrains inflammatory programs (Daignan-Fornier and Pinson, 2012). We also saw evidence of RNA modification/turnover changes. N^6^-methyladenosine (m^6^A), the dominant internal mRNA modification in plasma, was decreased. It is known to modulate stability/translation of inflammatory transcripts; emerging rheumatology work links m^6^A machinery to RA pathogenesis, so altered circulating m^6^A nucleosides are compatible with shifted RNA turnover during immune activation (Wu et al., 2021).

In contrast, 1-methylguanine was elevated. Modified purine bases/nucleosides (including 1-methylguanine/1-methylguanosine) are released during RNA turnover and are not efficiently salvaged; increases in biofluids are widely used as systemic markers of heightened RNA metabolism. The levels of the pyrimidine nucleoside, cytidine, were also observed to be reduced in patients with RA compared to controls. Beyond serving RNA synthesis, cytidine is consumed by activated cells and is regulated by cytidine deaminase. Clinical RA cohorts have previously shown elevated serum CDA activity, providing a plausible enzymatic route to lower circulating cytidine during active immune remodeling (Fatima et al., 2025; Gatarek et al., 2020). Taken together, an increase in succinyladenosine and 1-methylguanine coupled with a decrease in AICAR, inosine, m^6^A, and cytidine supports accelerated *de novo* purine synthesis and increased RNA turnover in RA. The relationship between m6A enzymes, immune cells, and RA suggests that m6A modification offers evidence for the pathogenesis of RA.

Patients with established disease or in remission are generally monitored using markers of inflammation, namely, ESR and CRP. An elevated ESR ≥ 28 mm/h and/or positive rheumatoid factor (RF) test are used to support the diagnosis of RA, although the levels for remission are lower (ESR < 20 mm/h for men and < 30 mm/h for women) (Wolfe and Michaud, 1994). As persistent systemic inflammation is expected to drive alterations in energy metabolism, amino acid turnover, lipid remodeling, and oxidative stress–related pathways observed in RA plasma profiles, stratification using ESR provides a biologically relevant variable for understanding the changes in metabolomic analysis. The subgroup analysis based on the stratification by ESR levels revealed significant dysregulation in a number of metabolites. From among these, levels of three plasma metabolites, namely, cysteinyl-serine, tyrosyl-arginine, and N2-acetyl, N6-methyllysine levels were observed to overlap with the significantly dysregulated metabolites identified in RA vs. control group. We observed that levels of cysteinyl-serine were increased both in patients with RA compared to controls and in patients with high ESR, suggesting a consistent change with both disease state and systemic inflammation. On the other hand, levels of tyrosyl-arginine and N2-acetyl, N6-methyllysine showed an opposite regulation, being increased in patients with RA compared to controls and were decreased in patients with high ESR levels. The dysregulation in the dipeptides cysteinyl-serine and tyrosyl-arginine point to alterations in systemic proteolysis consistent with the upregulation of cysteine and serine proteases in RA that increases chronic inflammation, which in turn is measured by ESR (Solau-Gervais et al., 2007). N2-acetyl, N6-methyllysine a doubly modified lysine is part of the lysine metabolic pathway, which has been reported to be associated with early RA patients who achieved remission. The increase in its levels in low ESR patients, points to a potential ongoing systemic inflammation, increased transcriptional activity and histone acetylation/methylation dynamics, activating an epigenetic activation/turnover signature in RA (Lu-Culligan et al., 2023). Similar to our results, previous studies also demonstrated an association between N2-Acetyl, N6-methyllysine with lower RA disease activity (Teitsma et al., 2018; Pinals et al., 1981). These metabolites together indicate a possible composite read of mitochondrial stress, proteolysis, and epigenetic reprogramming that converge on IL-6 and the hepatic acute-phase axis that influences chronic inflammation and ESR in patients with RA. On the other-hand, stratification with CRP did not reveal any metabolites that overlapped with the metabolites identified between RA and controls.

ROC analysis identified a panel of ten metabolites that showed the highest discriminatory power between RA and controls with an AUC of 0.993. These metabolites included succinyladenosine, PGP (24:0/PGD2), cysteinyl-serine, N-(1-Deoxy-1-fructosyl) phenylalanine, 2-(beta-D-Mannopyranosyl)-L-tryptophan, cysteinyl-alanine, CDP-DG (PGE2/i-19:0), CDP-DG (i-19:0/18:1 (12Z)-2OH(9,10)), L-Isoleucine and 4-Amino-1H-imidazole-2-carboxamide (Figure 4A). Bioinformatics and network Pathway analysis of the differentially regulated metabolites using the Ingenuity Pathway Analysis (IPA, QIAGEN), identified that the dysregulated the humoral immune response, inflammatory response, hematological system development and function pathways. The actions of these metabolites centered around regulating MAPK, PKC, IL6, IL1, and IL2, which are key inflammatory and signaling molecules, suggesting involvement in immune signaling, vascular function, and cytokine regulation consistent with previous studies (Ding et al., 2023). These findings are consistent with established RA pathophysiology, which is driven by interconnected TNF-α/NF-κB, IL-6/JAK–STAT, and MAPK signaling cascades that sustain chronic inflammation and immunometabolic dysregulation (Ucci et al., 2023). Recent studies have also identified Wnt signaling as an important immuno-inflammatory and metabolic regulator in RA, potentially intersecting with the dysregulated signaling networks identified in our metabolomic analysis (Riitano et al., 2025). The network pathway also showed the involvement of IL-21, a cytokine involved in stimulating follicular T helper cells, which was identified in our previous proteomic analysis (Masood et al., 2025). Canonical pathway analysis of the significantly altered metabolites revealed several biologically relevant processes associated with rheumatoid arthritis. Notably, G alpha (q) signaling events and Class A/1 (Rhodopsin-like receptors) pathways were significantly enriched and predicted to be inhibited in RA patients compared to controls. Aside from these pathways immune–metabolic dysregulation in RA has also been noted to be tightly coupled to epigenetic remodeling, dysregulated protein turnover, and sustained immune activation, which may contribute to the metabolite changes observed in the context of cellular stress and tissue remodeling (Riitano et al., 2023). Our results similarly show that these pathways are primarily involved in G-protein–coupled receptor signalling, which plays a critical role in immune cell activation, cytokine release, and inflammation. The observed inhibition suggests a possible disruption or downregulation of GPCR-mediated signalling in the RA metabolic landscape.

The findings from our study need to be interpreted in the context of our cohort that comprised of patients with long-standing rheumatoid arthritis receiving chronic therapy as part of their standard of care management that may confound the interpretation. Moreover, treatment variables were not included as covariates based on the heterogeneity within our cohort. Lastly our study employed an untargeted metabolomics approach aimed at exploratory and hypothesis generating allowing the identification of broad metabolic and pathway-level alterations in established RA. Future studies using larger cohorts and targeted analysis will be required to validate these findings.

## 5 Conclusion

Our plasma metabolomics analysis reveals that rheumatoid arthritis is characterized by coordinated changes across lipid, amino acid, nucleotide, and glycan metabolism, reflecting the biochemical imprint of persistent inflammation and immune metabolic dysregulation. Alterations in glycerophospholipids, acylcarnitines, dipeptides, nucleosides and their intermediates align with the fact that implies that autoimmune disorders like RA trigger multi-metabolite changes rather than singular alterations. These metabolite signatures not only deepen understanding of RA pathophysiology but also offer potential biomarkers for disease activity and treatment response, supporting the clinical value of metabolomics in precision patient stratification.

## Data Availability

The original contributions presented in the study are included in the article/Supplementary Material, further inquiries can be directed to the corresponding authors.

## Supplementary material

SUPPLEMENTARY FIGURE S1
**(A)** Metabolomics profiling between the high and low ESR subgroups within the RA. The volcano plot shows a significant change in the levels of several metabolites, of which red represents upregulated and blue represents downregulated tissue metabolites in RA and control groups (unpaired t-test, FDR q value ≤0.05, fold change ≥ 1.5). **(B)** Bar diagrams of the three metabolites in the high and low ESR subgroups within the RA * indicates significance <0.05 and **** indicates significance <0.0001.
