# Supplementary figures and images for: Untargeted metabolomics reveals immune-metabolic signatures in established cases of rheumatoid arthritis
Source: Front Mol Biosci. 2026 Feb 16;13:1755542. doi: 10.3389/fmolb.2026.1755542 (PMC12950679; doi:10.3389/fmolb.2026.1755542)

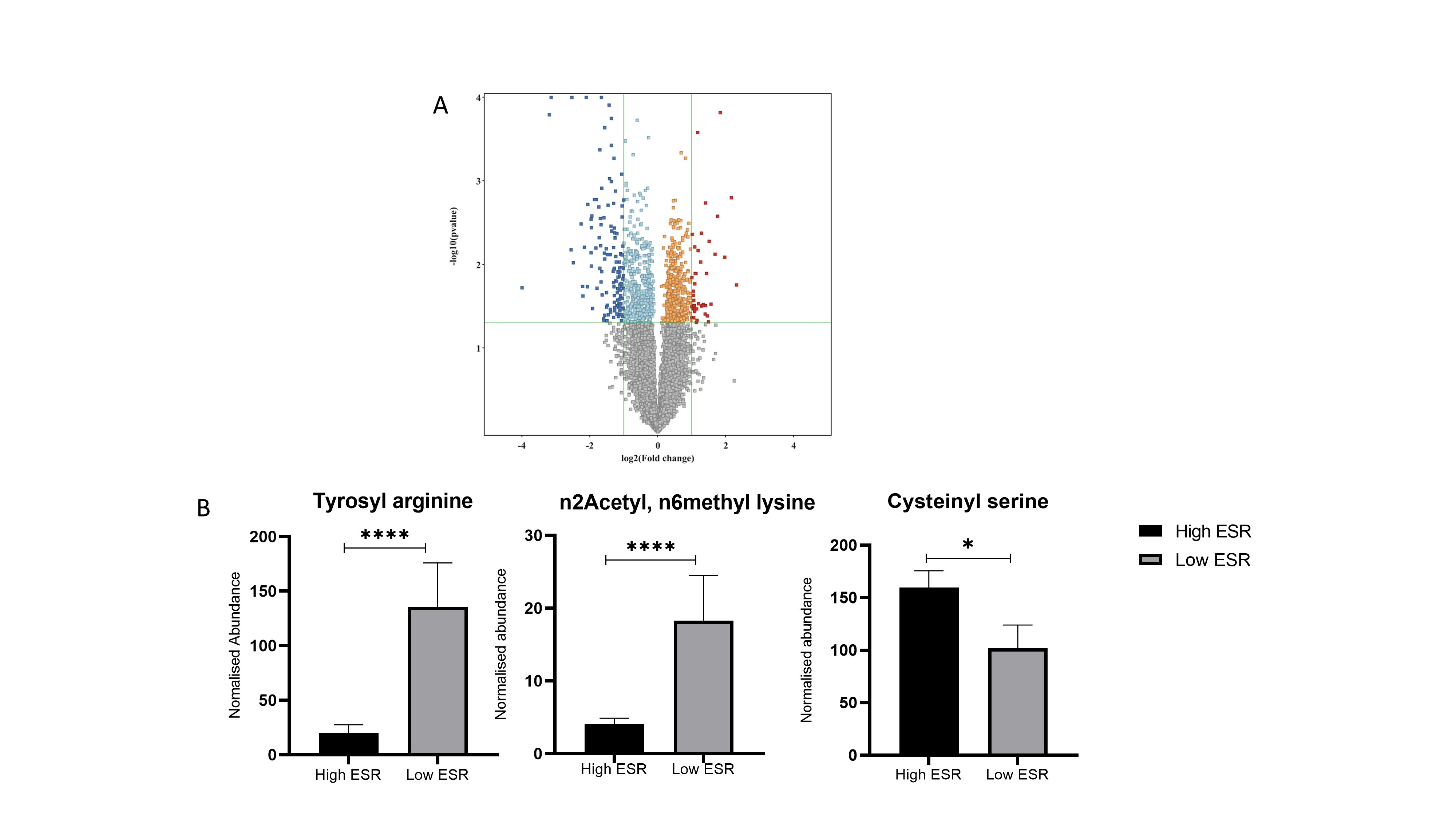

Supplement: Supplementary file 1 [file Image1.jpeg]
